# Supplementary material for: Mechanism of Arrhythmogenesis Driven by Early After Depolarizations in Cardiac Tissue
Source: PLoS Comput Biol. 2025 Apr 22;21(4):e1012635. doi: 10.1371/journal.pcbi.1012635 (PMC12047796; doi:10.1371/journal.pcbi.1012635)
Supplement: S1 Table — (DOCX) [file pcbi.1012635.s002.docx]

**Table S2****: Diffusion time scales**

| **Parameter** | **Description** | **Value** |
| --- | --- | --- |
| $\tau_{d}$ | Diffusion time between J and NJ cytosolic space | $5ms$ |
| $\tau_{d}^{sr}$ | Diffusion time between SR volumes between J and NJ sites | $5ms$ |
