# Supplementary material for: Mechanism of Arrhythmogenesis Driven by Early After Depolarizations in Cardiac Tissue
Source: PLoS Comput Biol. 2025 Apr 22;21(4):e1012635. doi: 10.1371/journal.pcbi.1012635 (PMC12047796; doi:10.1371/journal.pcbi.1012635)
Supplement: S1 Text — (DOCX) [file pcbi.1012635.s003.docx]

**S1 Text**

**Phenomenological model equations**

**Ca concentration equations**

To simulate the behavior of calcium (Ca) dynamics within the cell, we begin by noting that the cell's interior contains thousands of RyR2 (Ryanodine Receptor 2) clusters. These clusters can be categorized into two distinct groups. The first group consists of junctional clusters (J), where RyR2s are tightly linked to nearby L-type calcium channels (LCCs). The second group comprises non-junctional clusters (NJ), which are RyR2 clusters situated far from any local LCC cluster. Since these two populations respond differently to LCCs and we will treat them as distinct. These spaces are illustrated in Figure 1A and described in more detail in the main text. The total Ca concentration in each compartment, denoted with a superscript $T$, obeys the Ca flux equations:

$$\begin{aligned} v_{b}\frac{dc_{b}^{T}}{dt}=J_{r}^{b}-J_{up}^{b}-J_{Ca}+J_{NaCa}-J_{d}^{c},\#\left( 1 \right) \end{aligned}$$

$$\begin{aligned} v_{srb}\frac{dc_{srb}^{T}}{dt}=-J_{r}^{b}+J_{up}^{b}-J_{d}^{sr},\#\left( 2 \right) \end{aligned}$$

$$\begin{aligned} v_{i}\frac{dc_{i}^{T}}{dt}=J_{r}^{i}-J_{up}^{i}+J_{d}^{c},\#\left( 3 \right) \end{aligned}$$

$$\begin{aligned} v_{sri}\frac{dc_{sri}^{T}}{dt}={-J}_{r}^{i}+J_{up}^{i}+J_{d}^{sr},\#\left( 4 \right) \end{aligned}$$

The definition of each of these currents is given in Table 1 in the main text. For convenience we rescale the boundary and interior currents to the volume of the respective cytosol and make the replacements:

$$\begin{aligned} \frac{J_{r}^{b}}{v_{b}}\to J_{r}^{b} , \frac{J_{up}^{b}}{v_{b}}\to J_{up}^{b}, \frac{J_{NaCa}}{v_{b}}\to J_{NaCa}, \frac{J_{Ca}}{v_{b}}\to J_{Ca} , \frac{J_{d}^{c}}{v_{b}}\to J_{d}^{c} \#\left( 5 \right) \end{aligned}$$

$$\begin{aligned} \frac{J_{r}^{i}}{v_{i}}\to J_{r}^{i} , \frac{J_{up}^{i}}{v_{i}}\to J_{up}^{i} , \#\left( 6 \right) \end{aligned}$$

$$\begin{aligned} \left( \frac{1}{v_{srb}} \right)J_{d}^{sr}\to J_{d}^{sr} .\#\left( 7 \right) \end{aligned}$$

The equations are now written with the rescaled currents as

$$\begin{aligned} \frac{dc_{b}^{T}}{dt}=J_{r}^{b}-J_{up}^{b}-J_{Ca}+J_{NaCa}-J_{d}^{c},\#\left( 8 \right) \end{aligned}$$

$$\begin{aligned} \frac{dc_{srb}^{T}}{dt}=\left( \frac{v_{b}}{v_{srb}} \right)\left( -J_{r}^{b}+J_{up}^{b} \right)-J_{d}^{sr},\#(9) \end{aligned}$$

$$\begin{aligned} \frac{dc_{i}^{T}}{dt}=J_{r}^{i}-J_{up}^{i}+\left( \frac{v_{b}}{v_{i}} \right)J_{d}^{c} ,\#\left( 10 \right) \end{aligned}$$

$$\begin{aligned} \frac{dc_{sri}^{T}}{dt}=\left( \frac{v_{i}}{v_{sri}} \right)\left( {-J}_{r}^{i}+J_{up}^{i} \right)+\left( \frac{v_{srb}}{v_{sri}} \right) J_{d}^{sr}.\#\left( 11 \right) \end{aligned}$$

The diffusive fluxes between compartments are given by

$$\begin{aligned} J_{d}^{c}=\frac{c_{b}-c_{i}}{\tau_{d}} ,\#\left( 12 \right) \end{aligned}$$

$$\begin{aligned} J_{d}^{sr}=\frac{c_{srb}-c_{sri}}{\tau_{d}^{sr}},\#\left( 13 \right) \end{aligned}$$

where the concentrations are the free concentrations in the respective compartments, and where $\tau_{d}$ is the diffusion time scale linking J and NJ sites, and $\tau_{d}^{sr}$ is the time scale governing diffusion from corresponding SR volumes. Diffusion times are given in Table S1.

**The release flux from the SR**

The current flux from the SR due to J clusters will have the form

$$\begin{aligned} J_{r}^{b}=g_{b}c_{srb}p_{b}\left( t \right) ,\#\left( 14 \right) \end{aligned}$$

where $p_{b}\left( t \right)={n_{b}(t)}/{N_{b}}$. In this study we will not consider Ca release from NJ clusters so we will set $J_{r}^{i}=0$.

The SERCA uptake fluxes will have the form

$$\begin{aligned} J_{up}^{b}=g_{up}^{b}\frac{c_{b}^{H}}{{c_{b}^{*}}^{H}+c_{b}^{H}} ,\#\left( 15 \right) \end{aligned}$$

$$\begin{aligned} J_{up}^{i}=g_{up}^{i}\frac{c_{i}^{H}}{{c_{i}^{*}}^{H}+c_{i}^{H}} .\#\left( 16 \right) \end{aligned}$$

Here, $H$ is the Hill coefficient and $c_{b}^{*}$ and $c_{i}^{*}$ are the threshold for SERCA activation. Detailed parameters are listed on Table S2.

**The volume factors**

In order to solve the Ca flux equations it is necessary to determine the volume ratios given in Equations (9-11). To estimate these factors we will assume that in ventricular cells the total volume of J sites is double that of the N sites. Thus we will take $v_{b}=2v_{i}$. Similarly, we will take $v_{srb}=2 v_{sri}$. To determine volume ratios with the SR we follow Restrepo et al [1] who estimated that in ventricular myocytes the SR volume is roughly 30 times smaller than the cytosol. The ratio of cytosolic volume to SR volume will be taken to be ${v_{i}}/{v_{sri}}=30$, and ${v_{b}}/{v_{srb}=30}$.

**Buffers**

If $c_{x}^{T}$ denotes the total Ca concentration in compartment $x$, $c_{x}$ denotes the free concentration, $B$ is the total buffer concentration, and $[CaB]$ is the concentration of bound buffers then:

$$\begin{aligned} \frac{d\left[ CaB \right]}{dt}=k_{on}c_{x}\left( B-\left[ CaB \right] \right)-k_{off}\left[ CaB \right],\#\left( 17 \right) \end{aligned}$$

where $k_{on}$ and $k_{off}$ is the binding and dissociation rate respectively. For simplicity we assume instantaneous buffering so that the bound Ca is at steady state is

$$\begin{aligned} \left[ CaB \right]=\frac{Bc_{x}}{c_{x}+ K} ,\#\left( 18 \right) \end{aligned}$$

where $K=k_{on}/k_{off}$. Therefore, given the presence of multiple buffers with total concentration $B_{i}$ and kinetics $K_{i}$, the total Ca in the cell is given by

$$\begin{aligned} c_{x}^{T}=c_{x}+ \sum_{i} \frac{B_{i}c_{x}}{K_{i}+c_{x}} .\#\left( 19 \right) \end{aligned}$$

In this study we will apply two instantaneous cytosolic buffers. These are Calmodulin buffers with $B_{CaM}=24.0\mu M$ and $K_{CaM}=7.0$, and SR buffers with $B_{SR}=47.0\mu M$ and $K_{SR}=0.6$. Since Eq. (19) is nonlinear we will first fit the curve to a simpler function of the form

$$\begin{aligned} c_{x}^{T}=a_{1}c_{x}+\frac{a_{2}c_{x}}{a_{3}+c_{x}} .\#\left( 20 \right) \end{aligned}$$

Fitting the buffers for concentrations in the physiological range ${0.1\mu M\leq c}_{x}\leq5\mu M$ gives a solution $a_{1}=2.23895,a_{2}=52.0344, a_{3}=0.666509$. Inverting Eq. (20) yields the free concentration

$$\begin{aligned} c_{x}=\frac{1}{2a}\left( -a_{2}-a_{1}a_{3}+c_{x}^{T}+\sqrt{\left( a_{2}+a_{1}a_{3}-c_{x}^{T} \right)^{2}+4a_{1}a_{3}c_{x}^{T}} \right) .\#\left( 21 \right) \end{aligned}$$

In this approach Eqs. 8-11 are used to update the total Ca concentration in each compartment and Eq. 21 is used to determine the free concentrations from the total concentrations at each timestep.

**Spark rate parameters**

The spark recruitment rate at J sites is a function of the total Ca entry via $J_{Ca}$ and the average SR load. Thus, we will take the spark rate to have the form

$$\begin{aligned} \alpha_{b}=a_{b}{(P}_{O}+P_{OS})\left| i_{Ca} \right|\Phi\left( c_{srb} \right)\#\left( 22 \right) \end{aligned}$$

where $a_{b}$ is a constant, $P_{O}$ is the proability of being in the state $O$, $P_{OS}$ is the probability of being in the state $OS$, $i_{Ca}$ is the current through the LCC channel, and

$$\begin{aligned} \Phi\left( c_{srb} \right)=\frac{1}{1+\left( \frac{c_{srb}^{*}}{c_{srb}} \right)^{\gamma_{1}}} .\#\left( 23 \right) \end{aligned}$$

Model parameters are given in Table S3.

**The sodium-calcium exchange current**

In this study we use a standard formulation of $J_{NaCa}$

$$\begin{aligned} J_{NaCa}=A_{NaCa}\left( \frac{Na_{i}^{3}Ca_{o}\exp\left( 0.35z \right)-Na_{o}^{3}c_{b}\exp\left( \left( -0.65z \right) \right)}{\left( 1+0.2\exp\left( -0.65z \right) \right)U(c_{b})} \right)\#\left( 24 \right) \end{aligned}$$

where $z=VF/RT$, and where

$$\begin{aligned} A_{NaCA}=\frac{1}{1+\left( \frac{0.3}{c_{b}} \right)^{3}} ,\#\left( 25 \right) \end{aligned}$$

$$\begin{aligned} U(c)=K_{m,Cao}Na_{i}^{3}+K_{m,Nao}^{3}c_{b}+K_{m,Nai}^{3}Ca_{o}\left( 1+\frac{c}{K_{m,Cai}} \right) \\ +K_{m,Cai}Na_{o}^{3}\left( 1+\left( \frac{Na_{i}}{K_{m,Nai}} \right)^{3} \right)+Na_{i}^{3}Ca_{o}+Na_{o}^{3}c .\#\left( 26 \right) \end{aligned}$$

Model parameters used are: $K_{m,Cao}=1.3mM,K_{m,Cai}=0.0036mM, K_{m,Nai}=12.3mM, K_{m,Nao}=87.5mM.$ Concentration parameters are given in Table S4.

**The L-type Ca current**

We use a standard formulation of the LCC current. The driving force is given by

$$\begin{aligned} i_{Ca}=4P_{Ca}zF\frac{c_{b}\exp\left( 2z \right)-0.341Ca_{o}}{\exp\left( 2z \right)-1} ,\#\left( 27 \right) \end{aligned}$$

where $z={VF}/{RT}$. The open probability is governed my the Markov state diagram shown in Figure (1B) that is solved in the deterministic limit. The Ca independent transition rates are given by:

$$\begin{aligned} a_{12}=\frac{1}{1+\exp\left( -\frac{(V-1)}{7} \right)} ,\#\left( 28 \right) \end{aligned}$$

$$\begin{aligned} a_{21}=1-a_{12},\#\left( 29 \right) \end{aligned}\begin{aligned} r_{1}=0.3 , \#\left( 39 \right) \end{aligned}$$

$$\begin{aligned} a_{23}=0.3,\#\left( 30 \right) \end{aligned}$$

$$\begin{aligned} a_{32}=3 ,\#\left( 31 \right) \end{aligned}$$

$$\begin{aligned} a_{42}=0.00224, \#\left( 32 \right) \end{aligned}$$

$$a_{43}=a_{34}\left( \frac{a_{23}}{a_{32}} \right)\left( \frac{a_{42}}{a_{24}} \right),$$

$$\begin{aligned} P_{3}=\frac{1}{1+\exp\left( -\frac{V+40}{3} \right)}, \#\left( 33 \right) \end{aligned}$$

$$\begin{aligned} a_{45}=\frac{1-P_{3}}{3},\#\left( 34 \right) \end{aligned}$$

$$\begin{aligned} P_{r}=1-\frac{1}{1+\exp\left( -\frac{V+40}{4} \right)} , \#\left( 35 \right) \end{aligned}$$

$$a_{54}=a_{45}\left( \frac{a_{51}}{a_{15}} \right)\left( \frac{a_{24}}{a_{42}} \right)\left( \frac{a_{12}}{a_{21}} \right) ,$$

$$\begin{aligned} R=10+4954\exp\left( \frac{V}{15.6} \right) ,\#\left( 36 \right) \end{aligned}$$

$$\begin{aligned} \tau_{Ba}=\frac{\left( 1.5R-450 \right)P_{r}}{2}+225 ,\#\left( 37 \right) \end{aligned}$$

$$\begin{aligned} P=\frac{1}{1+\exp\left( -\frac{\left( V+40 \right)}{10} \right)} ,\#\left( 38 \right) \end{aligned}$$

$$\begin{aligned} a_{15}=\frac{P}{\tau_{Ba}},\#\left( 39 \right) \end{aligned}$$

$$\begin{aligned} a_{51}=\frac{1-P}{\tau_{Ba}} .\#\left( 40 \right) \end{aligned}$$

The Ca dependent transition rates are

$$\begin{aligned} a_{34}=0.00195+A_{Ca}F_{Ca}(c_{b}),\#\left( 41 \right) \end{aligned}$$

$$\begin{aligned} a_{24}=0.00413+{A_{Ca}F}_{Ca}(c_{b}),\#\left( 42 \right) \end{aligned}$$

where the Ca dependence is given by the term

$$\begin{aligned} F_{Ca}(c_{b})=\frac{1}{1+\left( \frac{c^{'}}{c_{b}} \right)^{2}} ,\#\left( 43 \right) \end{aligned}$$

and where $c^{'}=0.8\mu M$ is the diastolic Ca concentration. All transition rates between states facing a Ca spark are identical with the exception of the Ca transition rates, for which we set $F_{ca}=1$. This is because the Ca concentration in the vicinity of LCC channels during a Ca should be $\sim100\mu M$, which will saturate the Ca dependence of the LCC channel. The total open probability is given by the two components

$$\begin{aligned} P_{t}=P_{o}+P_{os} ,\#\left( 44 \right) \end{aligned}$$

so that we can write the total LCC current as $I_{Ca}=P_{t} \cdot i_{Ca}$.

**References**

1. Restrepo JG, Weiss JN, Karma A. Calsequestrin-mediated mechanism for cellular calcium transient alternans. Biophysical journal. 2008;95(8):3767-89.
