# Supplementary material for: Mechanism of Arrhythmogenesis Driven by Early After Depolarizations in Cardiac Tissue
Source: PLoS Comput Biol. 2025 Apr 22;21(4):e1012635. doi: 10.1371/journal.pcbi.1012635 (PMC12047796; doi:10.1371/journal.pcbi.1012635)
Supplement: S2 Table — (DOCX) [file pcbi.1012635.s004.docx]

**Table S1****: Ca cycling flux parameters**

| **Parameter** | **Description** | **Value** |
| --- | --- | --- |
| $g_{b}$ | Strength of Ca release from J clusters | $0.0067 \left( ms \right)^{-1}$ |
| $g_{i}$ | Strength of release from NJ sites | $0.0 \left( ms \right)^{-1}$ |
| $g_{up}^{b}$ | Boundary uptake strength | $0.4\mu M/ms$ |
| $g_{up}^{i}$ | Internal uptake strength | $0.4\mu M/ms$ |
| $H$ | SERCA Hill Coefficient | $3$ |
| $c_{b}^{*}$ | Boundary uptake threshold | $0.3\mu M$ |
| $c_{i}^{*}$ | Internal uptake threshold | $0.3\mu M$ |
| $g_{Ca}$ | L-type Ca current flux amplitude | $286\mu M\left( ms \right)^{-1}\left( pA \right)^{-1}$ |
| $g_{NaCa}$ | Sodium-Calcium exchanger flux amplitude | $1.5 \mu M\left( ms \right)^{-1}\left( pA \right)^{-1}$ |
