# Supplementary material for: Mechanism of Arrhythmogenesis Driven by Early After Depolarizations in Cardiac Tissue
Source: PLoS Comput Biol. 2025 Apr 22;21(4):e1012635. doi: 10.1371/journal.pcbi.1012635 (PMC12047796; doi:10.1371/journal.pcbi.1012635)
Supplement: S3 Table — (DOCX) [file pcbi.1012635.s005.docx]

**Table S3****: Spark rate parameters**

| **Parameter** | **Description** | **Value** |
| --- | --- | --- |
| $a_{b}$ | Spark rate at J clusters | $17.5 sparks/(ms\cdot pA)$ |
| $\gamma_{1}$ | Hill coefficient for SR load dependence of boundary spark rate | $10$ |
| $c_{srb}^{*}$ | Threshold for spark activation at junctional sites | $600\mu M$ |
| $\beta_{b}$ | Spark extinction rate at the cell boundary | $1/30 ms$ |
