# Supplementary material for: Mechanism of Arrhythmogenesis Driven by Early After Depolarizations in Cardiac Tissue
Source: PLoS Comput Biol. 2025 Apr 22;21(4):e1012635. doi: 10.1371/journal.pcbi.1012635 (PMC12047796; doi:10.1371/journal.pcbi.1012635)
Supplement: S4 Table — (DOCX) [file pcbi.1012635.s006.docx]

**Table S4: Constant parameters**

| Parameter | Description | Value |
| --- | --- | --- |
| $Na_{o}$ | External sodium concentration | $136mM$ |
| $Ca_{o}$ | External Ca concentration | $1.8mM$ |
| $K_{o}$ | External potassium concentration | $5.4mM$ |
| $K_{i}$ | Internal potassium concentration | $140mM$ |
| $T$ | Temperature | $308K$ |
| $F$ | Faraday's constant | $96.485C/mmol$ |
| $R$ | Universal gas constant | $8.315J\left( mol K \right)^{-1}$ |
| $P_{Ca}$ | LCC Permeability constant | $5.4\times{10}^{-4} cm/s$ |
