# Supplementary material for: Mechanism of Arrhythmogenesis Driven by Early After Depolarizations in Cardiac Tissue
Source: PLoS Comput Biol. 2025 Apr 22;21(4):e1012635. doi: 10.1371/journal.pcbi.1012635 (PMC12047796; doi:10.1371/journal.pcbi.1012635)
Supplement: Attachment — Submitted filename: response to reviewers.pdf [file pcbi.1012635.s008.pdf]

Reviewer's Responses to Questions

Reviewer #1

Comment

In this study, Stein and colleagues investigate the mechanisms underlying early afterdepolarizations (EADs) in cardiac tissue - first developing a phenomenological stochastic model that reproduces action potential duration (APD) variability, and then coupling cells in a 2D tissue. The authors demonstrate that the 2D tissue dynamics reproduce the deterministic limit of the stochastic cell cell model, which produces EAD alternans.

Overall, this is a rigorous and well-written study. I have only a few minor comments for the authors to address.

Response

We thank the referee for the overall positive review of our manuscript.

Comment

Clarification: For the 2D tissue data shown in Figure 4 and subsequent figures, does this also incorporate the reduced LCC inactivation, as in the single cell data shown in Figure 3? It seems as if this is the case, but not explicitly stated.

Response

All figures in Figure 4 correspond to the reduced LCC inactivation case.   Figure 4A-B shows the case where the system does not exhibit EADs, while Figure 4C-D shows the case with EADs.

In the revised manuscript we have clarified this by adding a new sentence on page 8 line 227 (red font)

Comment

Similarly, for the statistics (distributions and standard deviation measure) in tissue, do these measures include values for all points in space and in time? Or is APD first averaged across the tissue and then distribution based on time? Please clarify as applicable.

Response

In our simulations we have simply measured the APD for a cell at the approximate centre of the 2D tissue i.e. at location $(\lfloor N/2 \rfloor, \lfloor N/2 \rfloor)$. We made this choice since the APD is strongly correlated in space due to the electrotonic coupling. Thus, the APD of one cell is highly correlated with neighboring cells. Our simulations were relatively fast so we could afford to compute thousands of beats at just a single cell in the tissue.

In the revised manuscript we have clarified this point on page 8 (red font, line 227), and on the Figure 4 caption (red font).

Comment

Following the data in Figure 6C, D, how wide is the regime in which the "intermittent fluctuations" persist? I.e. How wide is the range between small fluctuations (shown in Figure 6A, B) and alternans (shown in Figure 6E, F)? It would be important to know if this behaviour is fairly robust, or a consequence of transitioning between these regimes.

Response

This is an excellent point. The intermittent fluctuations occur because the system is bistable and can transition from the stable fixed point to the period-2 solution. These two solutions are stable but still sensitive to noise so that system transitions between the two stable states. In the revised manuscript we have now included a full paragraph explaining in more detail the complex spatiotemporal dynamics that occurs during the bistable regime. This paragraph starts on page 12, line 355 (red font).

Comment

Additionally, the maximum spatial gradient may be another useful metric to quantify for these spatial simulations. It would be interesting to see if the spatial gradients are larger in the alternans regime or the intermittent fluctuations regime.

Response

This is an excellent question. A more detailed analysis of our simulations suggests that the spatial gradients in the intermittent and period-2 regimes are similar. However, we have not done a rigorous

analysis to make quantitative predictions. In the new manuscript we do mention that the spatial patterns are dynamic in the intermittent regime but remain static in the period-2 regime. We also state that the spatial patterns appear similar to the eye but a rigorous analysis of the emerging patterns have not been done in this study given the complexity of characterizing fluctuating spatiotemporal patterns. In the revised manuscript, we have added several sentences addressing this point at the end of the new paragraph on page 12 (line 355).

Comment

The demonstration of stable spatially discordant alternans in Figure 6F is interesting. It would be beneficial to discuss how this result at slow pacing rates differs from the more classical demonstration of spatially discordant alternans that arises at faster pacing rates and engages conduction velocity (CV) restitution. Presumably CV restitution is minimally engaged at these slow cycle lengths.

Response

This is an excellent point that we had not considered until this suggestion. The period-2 solution indeed occurs at longer cycle lengths; however, the amplitude of APD alternans is typically large, and after the long APD, the regime of small DI is engaged. Consequently, even at these long cycle lengths, the steep part of the conduction velocity (CV) restitution is still involved. In the revised manuscript, we emphasize that the shape of the APD restitution curve in Figure 8C induces large-amplitude APD alternans, which in turn engage the steep part of the CV restitution curve following the long action potential. This engagement is highly arrhythmogenic and serves as a driving mechanism for the wavebreak observed in Figure 7.

We have added new text at the end of page 15 (line 465, red font) to highlight that while this instability occurs at longer cycle lengths the regime of steep CV restitution is engaged.

Comment

Minor: The authors state that the wavebreaks shown in Figure 7 is robust and reliably occurs across multiple runs. Please provide an 'n' for the number of trials.

Response

A systematic and rigorous characterization of wavebreak in a paced stochastic system would require a more comprehensive statistical analysis. Here, we emphasize only that wavebreak was consistently observed across multiple simulation runs. We acknowledge that a detailed statistical study is necessary to fully characterize the system. On page 10 (line 296), we clarify this point and moderate our claim about robustness, stating instead that our observations suggest a possible mechanism.

Comment

The authors nicely comment on how the diffusion coefficient effectively determines the length scale of spatial coupling. Can the authors add to the discussion a bit to also highlight how reduced gap junctional coupling would also be expected to impact these results?

Response

This is an excellent point. In the revised manuscript, we emphasize that the spatial coupling length scale directly depends on a wide range of factors such as gap junction coupling and fibrosis. Thus, we expect that any factors that reduce gap junction coupling enhances fluctuations. We have added this clarification in the discussion at the end of page 11(line 345, marked red).

Reviewer #2

Comment

This study by Stein et al. investigates Early-after depolarizations (EADs) and their role in cardiac arrhythmia. Main results: Using a computational model, the researchers found that EADs, which vary randomly in single cells, become more synchronized in cardiac tissue due to gap junction coupling. They discovered that EADs emerge after a sudden transition to alternans, leading to synchronized EADs on every other beat. This transition, caused by a small change in pacing rate, is highly arrhythmogenic, promoting conduction block and reentry. The study highlights the critical role of EAD alternans in arrhythmogenesis, suggesting that ectopic beats are not necessary.

The manuscript is well-written, with a clear and commendable writing style. The discussion section is detailed, very interesting to read, and provides valuable insights into the study's implications.
The scope and findings of the study align well with the journal's focus, and the reviewer believes that the

manuscript will meet the high-quality standards expected by "PLOS Computational Biology" if a couple of concerns are addressed:

Response

We thank the reviewer for an overall positive assessment of our manuscript.

Comment

"has been modified for the ventricular cell geometry"
Has the model been modified only for cell geometry? Atrial cellular electrophysiology exhibits significantly different characteristics and may require distinct adjustments.

Response

The computational model presented here has been modified to account for ventricular cell geometry and we have used the main ionic currents in the Mahajan rabbit ventricular model. This is in contrast to our previous studies where we used the Grandi atrial cell model to model atrial cell electrophysiology. Indeed, EADs are sensitive to the underlying ionic model although they have been observed in both atrial and ventricular myocytes.

In the revised manuscript we explicitly state that our atrial model has been modified for both geometry and electrophysiology (page 3, line 76), and that we are using a ventricular cell model.

Comment

The (numerical) setup for the 2D simulations could be discussed in more detail in the methods section.

Response

In the revised manuscript we have rewritten the section on our 2D simulation methods and add more details about our computational methods. Page 6, line 164.

Comment

In the discussion, the authors address the length scale of electrical coupling and the differences that arise between 1D and 3D tissue. However, the simulations in this study were conducted only in 2D tissue.
I guess it would be feasible to perform simulations on a realistic 3D tissue patch, which would align more

closely with the focus of this part of the discussion. Open-source simulation frameworks such as openCMISS, openCARP, and Chaste are available and could facilitate such an approach. Do the authors anticipate any differences in their other findings if a proper 3D (finite element) tissue model was used instead of their 2D tissue representation?

Response

Indeed, we can apply all of our methods to 3D cardiac tissue by applying the openCARP simulation framework. However, we feel that a more thorough analysis of Ca-V in 3D will require a separate paper. Very likely geometrical effects will determine regions where EADs are prone to occur. Indeed, this is a topic of great interest to us given the recent evidence that the Purkinje-Ventricular junction is an important site for arrhythmia initiation.

In the revised manuscript we have now included a section in the discussion analyzing potential applications of our findings in the context of a 3D geometry. In particular we highlight a recent study showing that the ventricular-Purkinje junction plays a key role in arrhythmia initiation, and that a full 3D computational framework is needed to explore these effects. The new text is in page 16, line 477.

Comment

While the manuscript and the "Online Supplement" provide many equations and model parameters, I believe it would be challenging to fully replicate the results of this study, particularly the 2D setup.
Hence, I would not agree with the statement in the submitted file that
"All relevant data are within the manuscript and its Supporting Information files."
Notably, the computational code is neither mentioned nor made available in the study, which however seems to be required for submissions in PLOS Computational Biology:
"From the time of publication, Authors are required to make fully available and without restriction all data and computational code underlying their findings."
At a minimum, providing e.g. a CellML model implementation, like it is available for the mentioned Mahajan et al. model
(https://models.physiomeproject.org/exposure/5a23665da1793b4bfbad92defe7e639d), would be necessary to meet these requirements and ensure reproducibility.

Further, for computational studies like this it would be very helpful for reviewers to have a code framework available to repeat the simulations.
It is well known that there could be a large variability of results depending on choices on numerical

methods, see e.g. works of Pathmanathan et al. (https://doi.org/10.1002/cnm.2467) or Creswell et al. (https://doi.org/10.1098/rsif.2023.0369).
The equations presented in this paper appear sound. However, it is not possible for this reviewer to evaluate whether the chosen numerical framework is also appropriate. Thus it is challenging to assess if the main results mentioned above are justified.

Response

This is certainly an excellent and valid point. In the revised manuscript we have included a section on online data with a link to github so readers can download the code directly. Instruction on how to run the code and an explanation of model parameters are given in the github readme file.

Reviewer #3

Comment

Reviewer #3: The study is interesting, potentially very important, well-conducted, explained in detail and with substantial analysis and context. I don't have any major concerns about the key results or the analyses but do have some mechanistic questions and some minor issues that would need addressing.

Response

We thank the reviewer for a positive assessment of this paper.

Comment

Line numbers would always be helpful to identify specific statements.

Response

We have now turned on line numbers in our revised manuscript. All responses will refer explicitly to the line numbers.

Comment

Definition of EADs in the introduction omits any indication of a second upstroke in the AP. Are EADs in this case considered generally as prolonged APD (without or without an upward inflection)? Figures

illustrate that the EADs do contain an upstroke, so it may be more concise and accurate for the definition of EADs to reflect this.

Response

This is an excellent point. To our understanding EADs typically refer to the presence of one or more EAD upstrokes during the action potential. In our revised manuscript we have clarified the definition of EADs. This is shown in red font on line 39 (red font).

However, the upward deflection itself is not essential for the dynamical consequences. The critical response is a prolongation of the action potential (AP), which may occur without the characteristic EAD upstroke. In the revised manuscript, we clarify that our proposed mechanism for the period-2 bifurcation relies solely on a steep dependence of action potential duration (APD) on diastolic interval (DI). This steep relationship can arise even in the absence of EADs. See red font line 386.

Comment

I think it could be made clearer in the introduction the mechanism by which the stochasticity in calcium handling underpins EADs. Because it is debated whether a spontaneous release during an AP can directly elicit an EAD, which might be the obvious mechanism that comes to the reader’s mind, but this is not the mechanism investigated in this study, it would benefit to be clear about how the noise/stochasticity could underlie APD changes which then can lead to EADs by an ICaL mechanism (rather than a Jrel/NCX mechanism).

Response

This is an excellent and correct point. The EAD mechanism explored here relies only on ICaL and is independent of Ca waves that occur during the AP. In the revised manuscript we stress that the EAD mechanism is driven by IcaL, but that the stochasticity is due to the random recruitment of Ca sparks during the AP. Since Ca release is stochastic this induces APD fluctuations due to the sodium-calcium exchanger.

In the revised manuscript we explain with more clarity the source of the noise in our model. On line 177 we specify clearly that APD fluctuations are due to stochastic Ca spark recruitment which modulates the sodium-Calcium exchanger and thus the APD.

On line 314 we stress that our model for EADs does not consider the effect of Ca waves and depends solely on the L-type Ca current. Indeed, we mention that this can be a potential source of fluctuations but is not considered here.

Comment

I very much support the strategy to deal with LTCC inactivation differences depending on whether the channels are adjacent to sites of large local Ca or not.

Response

Yes, we have constructed our LCC model to distinguish between regions of high and low Ca. In our view LTCC inactivation can only be understood at the population level--where populations of channels either face low or high Ca.

Comment

Is the 2D tissue model isotropic or anisotropic? If isotropic, then could anisotropic coupling, where rather than an average it is comprised of strong (longitudinal) and weak (transverse) coupling, affect the results due to the different interactions of electrotonic load?

Response

Yes, the tissue models used in this study are isotropic, and we have not incorporated anisotropy. While anisotropy will influence the spatially discordant patterns by altering the electrotonic load, fully characterizing these effects is complex and requires a dedicated analysis. The suggestion to examine longitudinal and transverse differences is an excellent one, as discordant alternans is expected to be highly sensitive to both anisotropy and propagation direction.

To acknowledge this limitation, we have revised the discussion section to explicitly state that our model assumes isotropy and that anisotropy will play a significant role in shaping spatial patterns (Line 463, red font). We also mention the role of anisotropy in the 3D setting on line 482, and explain that these effects will dictate the sites of conduction block from the mechanism presented here.

Comment

I would like to see the statement of conduction velocities associated with the diffusion parameters used in the model (and how the CV varies with this variation in parameters and CL) so that we can better judge the electrotonic load conditions.

Response

In this study we have chosen a voltage diffusion coefficient of $D_V = 10^{-4} cm^2/ms$. This yields a conduction velocity on our 1D cable of roughly $10 cm/s$. We had picked a relatively low diffusion coefficient in order to model tissue effects within a reasonably sized 100X100 system. In the revised manuscript we have stated this CV in the methods section. We have also included an extensive discussion explaining that our results are highly sensitive to the effective diffusion coefficient and will depend strongly on gap junction coupling and fibrosis. The new text is on line 345 (red font).

Comment

I support the classification of APD at a voltage threshold to enable the consistent analysis in these complex situations. I suggest that the term "APD-40mV" could be used, analogous to "APD90" etc, so that it is always crystal clear which APD is being referred to, and to enable easy interpretation by readers who may miss this definition.

Response

This is an excellent idea. We have opted to use the notation APD_{-40mV} to indicate that the APD is measured at this voltage.

Comment

The authors discuss how their results regarding 1D strands are relevant for Purkinje induced arrhythmia, and this is certainly true and relevant. However, I think it is also worth noting that conditions such as increased fibrosis, connexin remodelling, ischemia etc could all lead to reduced coupling in tissue, including situations where 2D/3D tissue behaves more like loosely connected 1D strands, which would therefore have the same functional implications. This provides a more general context for how these mechanisms could be relevant in a range of disease conditions and may become more prominent during structural remodelling compared to health.

Response

Thank you for this insightful comment. In the revised manuscript, we have expanded our discussion on the factors influencing excitability. Additional text explaining the role of gap junction coupling, connexin remodeling and fibrosis has been included on page 11, starting at line 345 (highlighted in red).

Comment

Moreover, recent studies have highlighted other coupling mechanisms (e.g., ephaptic coupling) that could reduce electrotonic load during repolarisation, which may more readily enable EADs to appear (for example, this mechanism has been shown to more readily enable spatially discordant APD alternans). It may be worth mentioning this in the discussion in regards to the possibility that this present study underestimates the impact of EADs in 3D due to this overly high electrotonic load, and therefore that the mechanisms observed here may be even more relevant in general than is implied by the manuscript.

Response

This is an excellent point. Indeed, ephaptic coupling favors the formation of spatially discordant alternans and is highly relevant to this study. In the revised manuscript we have added new text explaining that the recent findings in ephaptic coupling, and that this effect will exacerbate the effects observed in this study. The new text is on line 350 (red font).

Comment

Moreover

9. In Figure 3, it could be very helpful to colour the corresponding AP traces in B using the red/black colour scheme used in A and C.

Response

In the revised figure we have shown the stable action potentials in black and the EAD action potentials in red. See the revised Figure 3B.

Comment

In Figure 6, I suggest that the scales of the y-axes in the left and right panels are set to be fully consistent, such that the ticks are aligned and that the scale of the variation in the right-hand panels visually directly

corresponds to that shown in the left-hand panels. I may also suggest to add the full label “beat n” rather than just “n” on the x-axis of the left panels.

Response

We have made these changes in the revised Fig. 6. The scales are now consistent between the left and right side.

Comment

A supporting information video of the case that led to re-entry would be very helpful to fully see this mechanism in action. At least, some more temporal snapshots within the paced beat that induces the re-entry would good. In general, it could be beneficial to show more 2D tissue panels that illustrate the spatial distribution of EADs (are they localised, whole tissue, centre of tissue only?)

Response

In the revised manuscript we have included a full movie showing the 2D evolution corresponding to Figure 7.   This movie is now included as an online supplement.
